# A Nomogram Prediction of Length of Hospital Stay in Patients with COVID-19 Pneumonia: A Retrospective Cohort Study

**DOI:** 10.1155/2021/5598824

**Published:** 2021-06-08

**Authors:** Kang Li, Chi Zhang, Ling Qin, Chaoran Zang, Ang Li, Jianping Sun, Yan Zhao, Yingmei Feng, Yonghong Zhang

**Affiliations:** Beijing You'An Hospital, Capital Medical University, Beijing, China

## Abstract

Assessing the length of hospital stay (LOS) in patients with coronavirus disease 2019 (COVID-19) pneumonia is helpful in optimizing the use efficiency of hospital beds and medical resources and relieving medical resource shortages. This retrospective cohort study of 97 patients was conducted at Beijing You'An Hospital between January 21, 2020, and March 21, 2020. A multivariate Cox proportional hazards regression based on the smallest Akaike information criterion value was used to select demographic and clinical variables to construct a nomogram. Discrimination, area under the receiver operating characteristic curve (AUC), calibration, and Kaplan–Meier curves with the log-rank test were used to assess the nomogram model. The median LOS was 13 days (interquartile range [IQR]: 10–18). Age, alanine aminotransferase, pneumonia, platelet count, and PF ratio (PaO_2_/FiO_2_) were included in the final model. The C-index of the nomogram was 0.76 (95%confidence interval [CI] = 0.69–0.83), and the AUC was 0.88 (95%CI = 0.82–0.95). The adjusted C-index was 0.75 (95%CI = 0.67–0.82) and adjusted AUC 0.86 (95%CI = 0.73–0.95), both after 1000 bootstrap cross internal validations. A Brier score of 0.11 (95%CI = 0.07–0.15) and adjusted Brier score of 0.130 (95%CI = 0.07–0.20) for the calibration curve showed good agreement. The AUC values for the nomogram at LOS of 10, 20, and 30 days were 0.79 (95%CI = 0.69–0.89), 0.89 (95%CI = 0.83–0.96), and 0.96 (95%CI = 0.92–1.00), respectively, and the high fit score of the nomogram model indicated a high probability of hospital stay. These results confirmed that the nomogram model accurately predicted the LOS of patients with COVID-19. We developed and validated a nomogram that incorporated five independent predictors of LOS. If validated in a future large cohort study, the model may help to optimize discharge strategies and, thus, shorten LOS in patients with COVID-19.

## 1. Introduction

Coronaviruses (CoVs) are a large family of single-stranded RNA viruses, and beta-CoVs have caused international outbreaks of emerging respiratory diseases, including severe acute respiratory syndrome coronavirus (SARS-CoV) in 2003 [1, 2] and Middle East respiratory syndrome-CoV (MERS-CoV) in 2012 [3]. In December 2019, a novel severe acute respiratory syndrome coronavirus 2 (SARS-CoV-2) infection in Wuhan led to coronavirus disease 2019 (COVID-19), with more than 290,000 confirmed cases in 174 countries and approximately 12,000 deaths (as of March 21, 2020) [4, 5]. The infectious disease outbreak led to a substantial increase in the demand for hospital beds, a shortage of medical equipment, and possible nosocomial infection among medical staff. According to the clinical condition of patients, physicians can evaluate the length of hospital stay (LOS), which is helpful in relieving medical resource shortages. A recent study reported a model including five variables, namely, procalcitonin, heart rate, Wuhan traveling history, lymphocyte count, and cough to predict prolonged LOS (>14 days) [6]. However, the model could only predict whether the LOS was >14 days. However, the “Wuhan traveling history” variable limited the extrapolative application of this model because the COVID-19 epidemic had been eliminated in Wuhan city.

We conducted a retrospective cohort study on the clinical characteristics of cured and discharged patients with confirmed COVID-19 infection between January 21, 2020, and March 21, 2020, in Beijing. We applied Cox proportional hazards regression to analyze time- (LOS-) to-event (discharge) data, which was able to provide individualized predictions of the estimated time to the event of interest. This study is aimed at describing the clinical characteristics of and develop and internally validate a predictive nomogram for estimating the LOS in patients with COVID-19.

## 2. Materials and Methods

### 2.1. Cohort Construction

This was a single-center, retrospective cohort study enrolling consecutive COVID-19 pneumonia patients aged over 18 years who underwent treatment at Beijing You'An Hospital between January 21, 2020, and March 21, 2020. All patients with COVID-19 pneumonia were diagnosed and classified according to the new coronavirus pneumonia diagnosis and treatment plan (trial version 6, in Chinese) developed by the National Health Committee of the People's Republic of China (http://www.nhc.gov.cn/). This study was approved by the Ethics Committee of Beijing You'An Hospital, and informed consent was obtained from all the patients.

### 2.2. Outcomes and Selection of Covariates

The primary outcome was LOS, which was defined as the time in days from hospital admission to discharge and was considered as “event =1” in Cox analysis. Readmission within two weeks was considered a prolonged LOS, and it was counted from the first hospitalization day. Death before discharge was also considered as a prolonged LOS and was estimated to be 800 days (longer than the longest LOS) and censored with “event = 0” in Cox analysis. Patients who died within 24 h of admission to the hospital were excluded from the Cox analysis. All patients were followed up for at least 6 months after discharge.

We collected baseline data, including demographic characteristics (age, sex, and comorbid diseases), epidemiological history, laboratory tests (biochemical indicators, routine blood testing, C-reactive protein, and chest radiograph or computed tomography [CT] scan), treatment, and outcome data. The data were extracted from the electronic medical record system, laboratory information system, and picture archiving and communication system.

### 2.3. Statistical Analysis

Continuous and categorical variables are presented as medians with interquartile ranges (IQRs) and *n* (%), respectively. We used Fisher's exact test or the chi-square test and the Mann–Whitney *U* test to make between-group comparisons of the subjects in the three groups. A backward stepwise method based on the smallest Akaike information criterion (AIC) value was applied to select covariates to be included in the Cox proportional hazards models.

The nomogram was developed using the “rms” *R* package. The area under the time-dependent receiver operating characteristic (ROC) curve was obtained using the “survival ROC” package. Harrell's C-index (concordance statistic, or C-statistic) was used to assess the predictive capacity of the nomogram. Bias-corrected calibration using the bootstrapping method with 1000 resamples was used for internal validation of the nomogram. Based on the scores of each variable, the total scores for each patient could be calculated using the “pec” package in *R*. The fit score of the five-covariate combination was used to stratify patients for Kaplan–Meier curve analysis using the log-rank test to compare the probability of hospital stay among the different groups, and the “survminer” package was applied in this regard. Statistical analyses were performed using *R* version 3.6.2. Extension packages, including “ggplot2,” “foreign,” and “export,” were also employed.

## 3. Results

### 3.1. Patient Population

**Figure 1:**
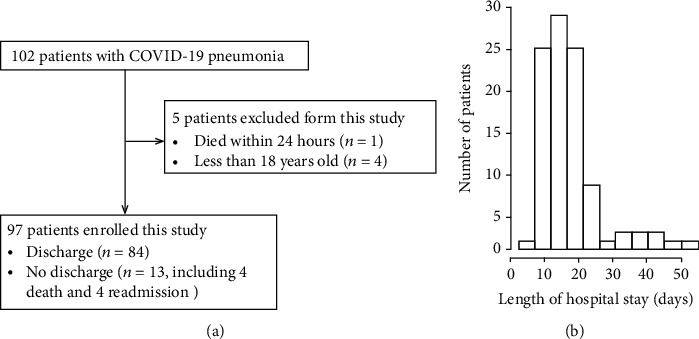
(a) Flow diagram of patient enrollment. (b) Distribution of length of hospital stay of discharged COVID-19 pneumonia patients.

A total of 102 patients were diagnosed with COVID-19 between January 21, 2020, and March 21, 2020, and treated at Beijing You'An Hospital. One patient who died within 24 h and four who were under 18 years of age were excluded from the analysis. Therefore, a total of 97 patients, including 84 (86.6%) discharged and 13 undischarged patients (including four deceased and four readmitted patients), were included in this study (Figure 1(a)). After at least 6 months of follow-up after discharge, there was no death. The baseline demographic characteristics of the study cohort are presented in Table 1. The median age of the study patients was 51.51 years (IQR: 38–64), and 42.3% were men. The primary outcome was LOS, and the median LOS was 13 days (IQR: 10–18). The LOS distribution of the discharged COVID-19 pneumonia patients is shown in Figure 1(b).

The LOS increased with age, and there was a significant difference among the three groups. The percentage of neutrophils, percentage of lymphocytes, platelet-to-lymphocyte ratio (PLR), and neutrophil-to-lymphocyte ratio (NLR) was significantly different among the three groups (all *p* < 0.01). The number of subjects with normal ALT and AST levels (both < 40 U/L) in the third group (LOS ≥ 19 days) was significantly lower than those in the other groups (*p* = 0.009 and *p* ≤ 0.001, respectively). Myoglobin and lactate levels in the third group (LOS ≥ 19 days) were significantly higher than those in the other groups (*p* ≤ 0.001 and *p* = 0.004, respectively).

### 3.2. Independent Predictors of LOS in Univariate and Multivariate Analysis

We assessed the LOS using Cox proportional hazard regression. Older age (≥50 years), high levels of ALT and AST (both ≥ 40 U/L), critical and severe pneumonia, and high levels of myoglobin (≥100 *μ*g/L) significantly increased the chance of longer LOS (all *p* < 0.05). In contrast, female sex, high platelet count (≥300 × 10^9^/L), high lymphocyte count (≥0.8 × 10^9^/L), high PF ratio (≥300 mmHg), and gradual increase in the glomerular filtration rate were significantly associated with shorter LOS (all *p* < 0.05). The other independent risk factors in the univariate analysis are shown in Table 2.

After backward elimination and model selection based on AIC, age (hazard ratio [HR] = 0.49; 95%confidence interval [CI] = 0.29–0.83, *p* = 0.00734), pneumonia (HR = 0.31, 95%CI = 0.18–0.52, *p* = 1.73*e* − 05), ALT (HR = 0.49, 95%CI = 0.29–0.83, *p* = 0.00697), PF ratio (HR = 1.45, 95%CI = 1.21 − 1.97, *p* = 0.0413), and platelet count (HR = 1.77, 95%CI = 0.93–3.39, *p* = 0.082) were included in the final model (smallest AIC value = 600.81) for the development of the nomogram (Table 2).

### 3.3. Development and Internal Validation of LOS-Predicting Nomogram

**Figure 2:**
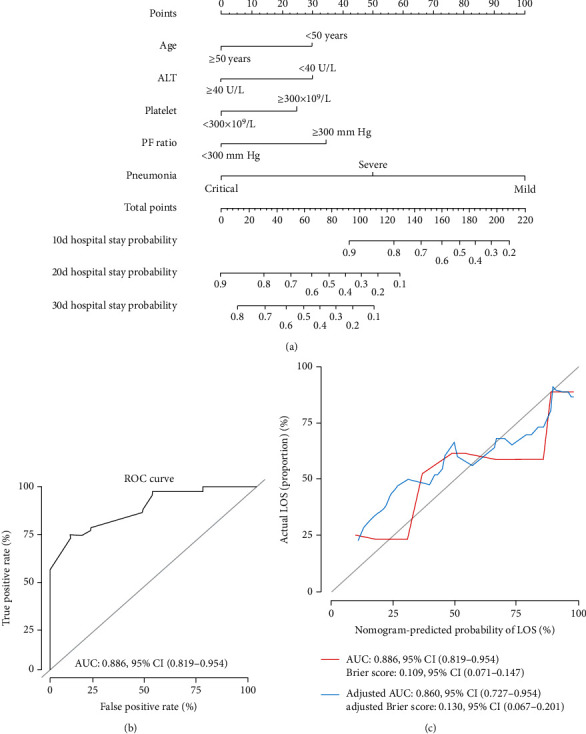
The nomogram and its predictive accuracy and discriminative ability. (a) Nomogram for the estimation of the probability of hospital stay of COVID-19 pneumonia patients. (b) Receiver operating characteristic curve of the nomogram. (c) The calibration curve showed favorable agreement between prediction by the nomogram and actual observations. The adjusted values were calculated by the bootstrap crossvalidation method, repeated 1000 times. ALT: alanine aminotransferase; PF ratio: PaO_2_/FiO_2_ ratio.

Five independently associated risk factors were used to form an LOS risk-estimating nomogram (Figure 2(a)). The nomogram demonstrated favorable accuracy in estimating the probability of hospital stay, with C-index values of 0.76 (95%CI = 0.69–0.83) and AUC of 0.88 (95%CI = 0.82–0.95) (Figure 2(b)). The overfit of the model was estimated by applying the bootstrap internal validation method. The adjusted C-index was 0.75 (95%CI = 0.67–0.82) and adjusted AUC 0.86 (95%CI = 0.73–0.95) after 1000 bootstrap crossvalidation iterations (Figure 2(c)), which represented the bias-corrected estimate of model performance in the future and demonstrated favorable predictive accuracy for the nomogram. A Brier score of 0.11 (95%CI = 0.07–0.15) and adjusted Brier score of 0.13 (95%CI = 0.07–0.20) for the calibration curve demonstrated favorable agreement between prediction probability by nomogram and actual state of hospitalization (Figure 2(c)).

**Figure 3:**
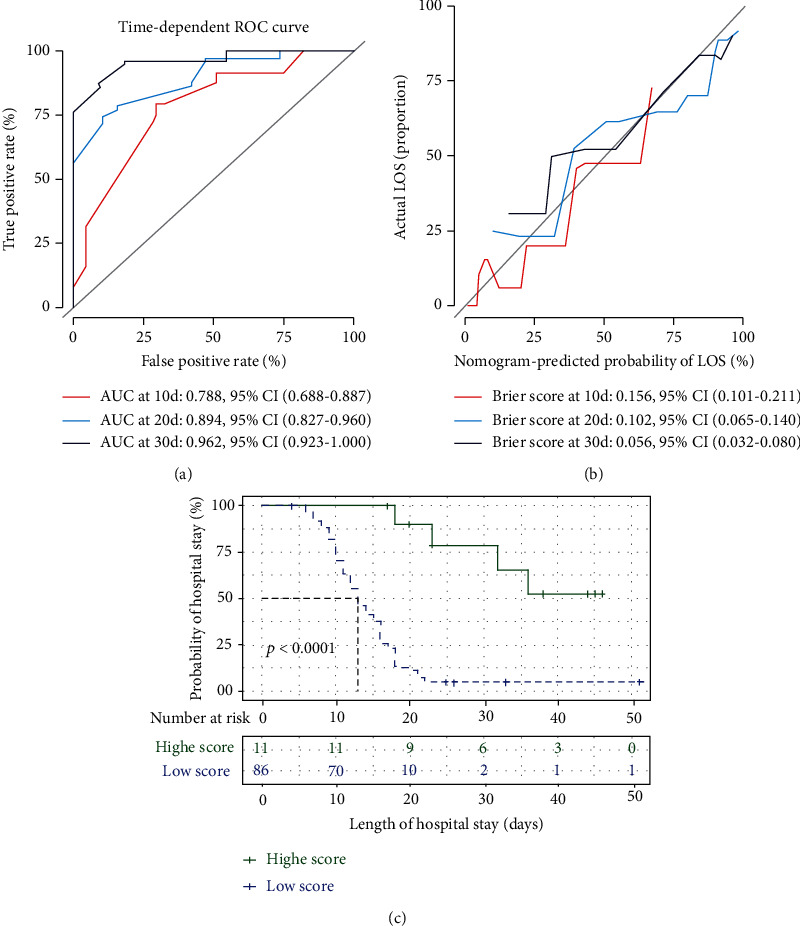
Time-dependent receiver operating characteristic curve showing area under curve (AUC) values at 10 (red), 20 (blue), and 30 (black) days (a). The Brier score of the calibration curve for the nomogram at 10 (red), 20 (blue), and 30 (black) days (b). Kaplan–Meier curves comparing the probability of hospital stay among the different patient groups, stratified by the fit score of the five-covariate nomogram model (c). *p* values were calculated using the log-rank test.

Finally, the area under the time-dependent ROC curve was used to validate the ability of the nomogram to discriminate patients who were discharged within 10, 20, and 30 days of hospital stay. The AUC values for the nomogram at 10, 20, and 30 days were 0.79 (95%CI = 0.69–0.89), 0.89 (95%CI = 0.83–0.96), and 0.96 (95%CI = 0.92–1.00), respectively (Figure 3(a)). The Brier score of the calibration curve for the nomogram at 10, 20, and 30 days was 0.16 (95%CI = 0.10–0.21), 0.10 (95%CI = 0.07–0.14), and 0.06 (95%CI = 0.03–0.08), respectively (Figure 3(b)). The Kaplan–Meier curves together with the log-rank test also demonstrated that a high fit score nomogram model indicated a high probability of long hospital stay in the training group (Figure 3(c), log-rank *p* < 0.0001). These results confirmed that the nomogram model accurately predicted the LOS of patients with COVID-19.

## 4. Discussion

COVID-19 has emerged as a worldwide pandemic; at present, the number of infected people continually increases substantially every day in most countries of the world. According to patient clinical data, physicians can evaluate their length of stay. It is beneficial to optimize the use efficiency of hospital beds and medical resources and relieve medical resource shortages.

In this retrospective cohort study, we found that the median LOS was 13 days (IQR: 10–18). Age, ALT, PF ratio, pneumonia, and platelet count were independently associated with LOS in patients with COVID-19, and they were included in the final nomogram. The prognostic model demonstrated a significantly higher predictive accuracy and discriminative ability for the prediction of 10-, 20-, and 30-day LOS for COVID-19-infected patients. Further, the nomogram demonstrated favorable discrimination and superior performance in internal validation. The nomogram model with a high fit score indicated a high probability of hospital stay. These results confirmed that the nomogram model accurately predicted the LOS of patients with COVID-19.

Older age is an important independent predictor of mortality [7]. Similar results were obtained for SARS [1, 8] and MERS [9]. Both cell-mediated immunity and humoral immune function evidently declined in elderly patients. Concomitantly, cytokine and chemokine signaling networks in elderly patients changed; type 2 cytokine response tended to be more sensitive than type 1 [10], and the proportion of T cells producing IL-4, IL-8, and IL-10 increased with age [11]. In these cases, viral replication and longer-lasting proinflammatory responses were not controlled. In SARS-CoV and MERS-CoV infection, uncontrolled induction of proinflammatory cytokines resulted in pathogenesis and disease severity [12]. Several days after COVID-19 infection, patients presented symptoms such as fever, coughing, sputum, vomiting, and diarrhea, and they were diagnosed and treated in the hospital. Fever (≥37.3°C) was an initial important event integral to immune response [13]; however, it was not significantly associated with LOS in univariate analysis.

Platelets are part of the first line of defense against lung-specific entry of SARS-CoV-2 [14], and among patients who had the lowest platelet counts, mortality decreased with an increase in platelet count [15]. The improvement in platelet count might have indicated clinical improvement. Monitoring of platelet counts is certainly beneficial to clinicians in rare resource environments, where the chance of laboratory examination may be limited; however, the whole blood count may be relatively easy [15, 16].

Acute respiratory distress syndrome (ARDS), characterized by hypoxemia with a PaO_2_/FiO_2_ ratio (P/F ratio) ≤ 200 mmHg, is the primary cause of death due to COVID-19. ARDS is a heterogeneous clinical syndrome, which is mechanically induced by uncontrolled COVID-19 viral replication and host cytokine storm. COVID-19 has unique ARDS characteristics in medical imaging and has been reported as a variable in several diagnostic studies. Artificial intelligence is a diagnostic tool that combines multiple imaging modalities, including lung CT, chest radiography, and lung ultrasound [17]. Accordingly, AI assisted us to comprehensively interpret clinical and multiomics data of ARDS patients, and it is potentially advantageous in the management of ARDS patients in the future with individual treatment plans [18].

There are certain limitations to our study. First, this was a single-center, retrospective cohort study involving approximately a quarter of the COVID-19 patients in Beijing on March 21, 2020. This was not representative of the overall COVID-19 treatment or LOS in this area. Second, owing to low mortality (5/102), this study could not analyze the risk factors for survival. Third, due to the retrospective cohort design, laboratory tests were not performed for all cytokines. For example, interferon-inducible protein-10 and IL-6 are predictive factors for SARS [19] and COVID-19 [7] outcomes, respectively; yet, they were excluded.

## 5. Conclusions

We successfully developed and validated a nomogram, which incorporated five independent predictors of LOS. Provided a future, large sample size cohort study that is used to validate the model, it may be useful in optimizing discharge strategies, hence shortening LOS in patients with COVID-19.

## Figures and Tables

**Table 1:** Summary statistics of patient demographics and clinical characteristics (by quartile of LOS).

	Total	≤10 days (*n* = 26)	11–18 days (*n* = 49)	≥19 days (*n* = 22)	*p* value
Demographic characteristics					
Age, years (IQR)	51.5 (38–64)	41.0 (31,62.5)	49 (37–60)	63 (57–74.8)	≤0.001^∗∗∗^
Male sex, *n* (%)	41 (42.3)	9 (34.6)	18 (36.7)	14 (63.6)	0.069
Clinical findings					
Pneumonia, *n* (%)					
Mild	69 (71.1)	23 (88.5)	39 (79.6)	7 (31.8)	≤0.001^∗∗∗^
Severe	17 (17.5)	3 (11.5)	8 (16.3)	6 (27.3)	
Critical	11 (11.3)	0 (0.0)	2 (4.1)	9 (40.9)	
Fever (°C), *n* (%)					
<37.3	24 (24.7)	11 (42.3)	11 (22.4)	2 (9.1)	0.045^∗^
37.3–38.5	50 (51.5)	13 (50.0)	24 (49.0)	13 (59.1)	
>38.5	23 (23.7)	2 (7.7)	14 (28.6)	7 (31.8)	
Cough	58 (59.8%)	13 (46.4%)	31 (62%)	15 (68.2%)	0.423
Sputum	25 (25.8%)	5 (19.2%)	12 (24.5%)	8 (36.4%)	0.384
Vomiting	4 (4.1%)	1 (3.8%)	2 (4.1%)	1 (4.5%)	0.993
Diarrhea	2 (2.1%)	1 (3.8%)	0 (0%)	1 (4.5%)	0.238
Lung CT^(a)^	85 (87.6%)	21 (80.8%)	44 (91.8%)	19 (86.4%)	0.384
Coexisting illnesses					
Kidney disease	3 (3.1%)	1 (3.80%)	2 (4.1%)	0 (0.0%)	0.455
Hypertension	22 (22.7%)	2 (15.4%)	9 (18.4%)	9 (40.9%)	0.081
Hyperlipidemia	3 (3.1%)	1 (3.8%)	1 (2.0%)	1 (4.5%)	0.825
Diabetes	8 (8.2%)	2 (7.7%)	4 (8.2%)	3 (13.6%)	0.984
Heart disease	10 (10.3%)	2 (7.7%)	5 (10.2%)	2 (10.5%)	0.798
Lung disease	7 (7.2%)	2 (7.7%)	4 (8.2%)	1 (4.5%)	0.844
Surgery	22 (22.7%)	4 (15.4%)	12 (24.5%)	6 (27.3%)	0.564
Laboratory indicators					
White blood cell count, ×10^9^/L	4.4 (3.5–5.9)	4.1 (3.5–5.8)	4.4 (3.5–5.7)	5.0 (3.2–6.9)	0.583
<4	40 (41.2%)	12 (46.2%)	20 (40.8%)	8 (36.4%)	0.787
≥4	57 (58.8%)	14 (53.8%)	29 (59.2%)	14 (63.6%)	
Hemoglobin, g/dL	136 (125–144)	131 (120.3–144.3)	135 (126–143.5)	139 (129–149)	0.157
Platelet count × 10^9^/L	194 (160–238)	206 (161–298.3)	193 (144.5–245)	191 (147.5–226.3)	0.257
<100	5 (5.2%)	0 (0.0%)	2 (4.1%)	5 (10.2%)	0.071
100–300	80 (82.5%)	20 (76.9%)	42 (85.7%)	6 (23.1%)	
>300	12 (12.4%)	6 (23.1%)	5 (10.2%)	1 (4.5%)	
Lymphocyte count × 10^9^/L	1.1 (0.77–1.53)	1.26 (0.93–1.61)	1.18 (0.80–1.54)	0.81 (0.70–1.21)	0.026^∗^
<0.8	26 (26.8%)	5 (19.2%)	11 (22.4%)	10 (45.5%)	0.90
≥0.8	71 (73.2%)	21 (80.8%)	38 (77.6%)	12 (54.5%)	
Monocyte count×10^9^/L	0.31 (0.21–0.44)	0.34 (0.22–0.42)	0.25 (0.16–0.39)	0.32 (0.19–0.51)	0.928
Neutrophil count × 10^9^/L	2.69 (1.84–4.09)	2.37 (1.86–3.42)	2.69 (1.84–3.83)	3.71 (1.68–5.38)	0.067
<1.8	21 (21.6%)	5 (19.2%)	10 (20.4%)	6 (27.3%)	0.206
1.8–6.3	70 (72.2%)	21 (80.8%)	36 (73.5%)	13 (59.1%)	
>6.3	6 (6.2%)	0 (0%)	3 (6.1%)	3 (13.6%)	
Lymphocyte percentage	26.1 (17.9–34.5)	33 (21.7–37.8)	26.1 (19.3–34.3)	18.7 (19.3–34.5)	0.002^∗∗^
<20	29 (29.9%)	3 (11.5%)	14 (28.6%)	12 (54.5%)	0.007^∗∗^
20–40	58 (59.8%)	19 (73.1%)	29 (59.2%)	10 (45.5%)	
>40	10 (10.3%)	4 (15.4%)	6 (12.2%)	0 (0.0%)	
Neutrophil percentage	64.0 (51.8–72.3)	55.4 (49.2–69.1)	64 (53.1–72.7)	70.4 (60.4–79.6)	0.002^∗∗^
<75	78 (80.4%)	25 (96.2%)	39 (79.6%)	14 (63.6%)	0.018^∗^
≥75	19 (19.6%)	1 (3.8%)	10 (20.4%)	8 (36.4%)	
NLR	2.4 (1.4, 3.9)	1.7 (1.3–3.1)	2.3 (1.6–3.8)	3.61 (1.8–6.3)	0.004^∗∗^
<2.75	56 (57.7%)	18 (69.2%)	31 (63.3%)	7 (31.8%)	0.018^∗^
2.75	41 (42.3%)	8 (30.8%)	18 (36.7%)	15 (68.2%)	
LMR	3.7 (2.8, 5.3)	4.1 (2.9, 6.3)	3.9 (2.8, 5.4)	3.0 (2.3,4.8)	0.268
<2.63	20 (20.8%)	5 (19.2%)	7 (14.3%)	8 (38.1%)	0.096
≥2.63	76 (79.2%)	21 (80.8%)	42 (85.7%)	8 (61.9%)	
PLR	177.3 (124.8–246.2)	132.7 (118.5–172.3)	188.1 (124.8–156.6)	221.5 (163.6–182.5)	0.018^∗^
<160	41 (42.3%)	15 (57.7%)	21 (42.9%)	5 (22.7%)	0.045
≥160	56 (57.7%)	11 (42.3%)	28 (57.1%)	17 (77.3%)	
Prothrombin time, s	12.6 (12.1–131.1)	12.8 (12.2–13.4)	12.4 (11.9–12.87)	12.75 (12.1–13.42)	0.417
Prothrombin activity, percentage	75 (71–80)	73.5 (68.5–79.0)	76.0 (73.0–82.0)	74.0 (68.5–78.5)	0.459
<75	46 (48.4%)	15 (57.7%)	19 (39.6%)	12 (57.1%)	0.219
≥75	49 (51.6%)	11 (43.3%)	29 (60.4%)	9 (42.9%)	
C-reactive protein, mg/L	14.7 (3.4–37.4)	12.7 (2.0–18.3)	16.8 (3.3, 41.15)	19.4 (10.0–54.13)	0.314
Procalcitonin, ug/L	0.11 (0.10–0.14)	0.10 (0.08–0.15)	0.11 (0.06, 0.15)	0.12 (0.10–0.14)	0.256
Fibrinogen, g/L	3.2 (2.5–4.3)	3.0 (2.5–4.3)	3.2 (2.5–4.1)	3.2 (2.5–4.4)	0.549
ALT, U/L	28 (20–45)	26.5 (20–39)	26 (20–42)	42 (19.7–52.7)	0.126
<40	65 (67.0%)	21 (80.8%)	35 (71.4%)	9 (40.9%)	0.009^∗∗^
≥40	32 (33.0%)	5 (19.2%)	14 (28.6%)	13 (59.1%)	
AST, U/L	30 (21.5–42)	25.5 (20.5–34)	28.0 (21–40)	42.5 (22.75–64.5)	0.004^∗∗^
<40	70 (72.2%)	24 (92.3%)	38 (77.6%)	3 (36.4%)	≤0.001^∗∗∗^
≥40	27 (27.8%)	2 (7.7%)	11 (22.4%)	14 (63.6%)	
AST/ALT	1.04 (0.76–1.35)	0.96 (0.60, 1.43)	1.02 (0.73–1.30)	1.30 (0.89, 1.71)	0.108
Total bilirubin, mmol/L	9.60 (7.10–13.05)	8.80 (6.13–12.10)	9.20 (6.80–12.60)	12.35 (9.38–14.75)	0.046^∗^
Albumin, g/L	36.8 (33–39.8)	37.60 (33.8–40)	36.6 (33.4–39.9)	36.2 (32.5–40)	0.158
<35	36 (37.1%)	8 (30.8%)	17 (34.7%)	11 (50.0%)	0.350
≥35	61 (62.9%)	18 (69.2%)	32 (65.3%)	11 (50.0%)	
Glomerular filtration rate, (mL/min)	99.5 (91–113.75)	109.35 (94.8–119.7)	105.3 (94.2–117.6)	94.5 (80.3–97.4)	0.001^∗∗^
Carbon dioxide combining power, mmol/L	26.8 (24.4–28.9)	25.70 (24.4–28.3)	27.2 (24.7–28.9)	27 (23–28.9)	0.428
Creatine kinase, U/L	72 (46–118)	59 (42–100.3)	72 (46–118)	118 (59.3–353)	0.203
<185	83 (85.6%)	25 (96.2%)	43 (87.8%)	15 (68.2%)	0.022^∗^
≥185	14 (14.4%)	1 (3.8%)	6 (12.2%)	7 (31.8%)	
Creatine kinase isoenzymes, CK-MB, ng/mL	0.34 (0.16–0.73)	0.29 (0.08–0.61)	0.28 (0.13–0.69)	0.57 (0.27–1.10)	0.008^∗∗^
<5	66 (68.0%)	17 (65.4%)	39 (79.6%)	10 (45.5%)	0.016^∗∗^
≥5	31 (32.0%)	9 (34.6%)	10 (20.4%)	12 (54.5%)	
Myoglobin, *μ*g/L	45 (30–66)	34 (27.5–50)	38 (29–60.5)	66 (49.5–187.5)	≤0.001^∗∗∗^
<100	84 (86.6%)	24 (92.3%)	46 (93.9%)	14 (63.6%)	0.004^∗∗^
≥100	13 (13.4%)	2 (7.7%)	3 (6.1%)	8 (36.4%)	
Lactate, mmol/L	1.2 (0.9–1.7)	1.0 (0.9–1.2)	1.24 (0.9–1.7)	1.6 (1.2–1.9)	0.004^∗∗^
<1.7	69 (73.4%)	22 (88.0%)	37 (78.7%)	10 (45.5%)	0.02^∗^
≥1.7	25 (26.6%)	3 (12.0%)	10 (21.3%)	12 (54.5%)	
PF ratio, mmHg	433.5 (311.4–527.4)	471.3 (293.5–530.8)	446 (370.8–572.9)	340 (223.4–447.4)	0.02^∗^
<300	20	7	4	9	0.003^∗∗^
≥300	77	19	45	13	
Treatment					
Antibiotics	30 (30.9%)	11 (42.3%)	13 (26.5%)	6 (27.3%)	0.340
Antiviral treatment	37 (38.1%)	10 (38.5%)	19 (38.8%)	8 (36.4%)	0.981
Chinese medicine treatment	74 (76.3%)	22 (84.6%)	36 (73.5%)	16 (72.7%)	0.505
Corticosteroids	19 (19.6%)	0 (0.0%)	8 (16.3%)	11 (50.0%)	≤0.001^∗∗∗^
Oxygen therapy	34 (35.1%)	4 (15.4%)	19 (38.8%)	11 (50.0%)	0.032^∗^
Ventilator	6 (6.2%)	0 (0.0%)	2 (4.1%)	4 (18.2%)	0.024^∗^

^(a)^Positive result: CT images showing multiple patchy ground-glass opacities along the peribronchial and subpleural lungs; NLR: neutrophil-to-lymphocyte ratio; LMR: lymphocyte-to-monocyte ratio; PLR: platelet-to-lymphocyte ratio; ALT: alanine aminotransferase; AST: aspartate aminotransferase; PF ratio: PaO_2_/FiO_2_ ratio. Significance codes: “^∗∗∗^”0.001, “^∗∗^”0.01, “^∗^”0.05.

**Table 2:** Prognostic factors associated with LOS in COVID-19 pneumonia.

Variables	Univariate		Multivariate	
	HR (95% CI)	*p* value	HR (95% CI)	*p* value
Age, years (≥50 vs. < 50)	0.58 (0.33–1.01)	3.22*e*-04^∗∗∗^	0.49 (0.29–0.83)	0.00734^∗∗^
Sex (female vs. male)	1.89 (1.71–3.25)	0.0077^∗^		
ALT, U/L (≥40 vs. <40)	0.45 (0.33–0.87)	0.0065^∗^	0.49 (0.29–0.83)	0.00697^∗∗^
AST, U/L (≥40 vs. <40)	0.42 (0.23–0.78)	3.34*e*-03^∗∗^		
Fever (°C) (≥37.3 vs. < 37.3)	0.58 (0.35–1.08)	0.39		
Pneumonia (critical + severe vs. mild)	0.33 (0.049–0.47)	1.52*e*-03^∗∗^	0.31 (0.18–0.52)	1.73*e*-05^∗∗∗^
Hemoglobin, g/L (per unit)	0.97 (0.96–0.99)	0.034^∗^		
Lymphocyte count × 10^9^/L (≥0.8 vs. < 0.8)	1.55 (0.78–2.51)	0.026^∗^		
Neutrophil count × 10^9^/L (per unit)	0.96 (0.84–1.16)	0.047^∗^		
Platelet count ×10^9^/L (≥300 vs. <300)	2.52 (0.69–7.55)	0.012^∗^	1.77 (0.93–3.39)	0.08201
NLR (≥2.75 vs. < 2.75)	0.77 (0.66–1.20)	0.22		
C-reactive protein, mg/L (≥2.2 vs. < 2.2)	0.57 (0.33–1.15)	0.09.		
PLR (≥160 vs. < 160)	0.68 (0.54–0.88)	0.478		
Albumin (g/L) (≥35 vs. < 35)	0.79 (0.49–1.58)	0.692		
GFR (mL/min) (per unit)	1.12 (1.11–1.13)	0.021^∗^		
Creatine kinase, U/L (≥185 vs. < 185)	0.62 (0.36–1.22)	0.152		
Creatine kinase isoenzymes MB, ng/mL) (≥5 vs. < 5)	1.46 (0.81–2.88)	0.447		
Myoglobin, *μ*g/L (≥100 vs. < 100)	0.21 (0.12–0.76)	1.65*e*-03^∗∗^		
Lactate, mmol/L (≥1.7 vs. < 1.7)	0.77 (0.26–1.22)	0.194		
PF ratio, mmHg (≥300 vs. < 300)	1.75 (0.99–3.12)	0.0405^∗^	1.45 (1.21–1.97)	0.04133^∗^

ALT: alanine aminotransferase; AST: aspartate aminotransferase; NLR: neutrophil-to-lymphocyte ratio; PLR: platelet-to-lymphocyte ratio; GFR: glomerular filtration rate; PF ratio: PaO2/FiO2 ratio. Significance codes: “^∗∗∗^”0.001, “^∗∗^”0.01, “^∗^”0.05, “.”0.1, “ ”1.

## Data Availability

The data used to support the findings of this study are included within the article.
